# Hereditary Aortic Aneurysms and Dissections: Clinical Diagnosis and Genetic Testing

**DOI:** 10.3400/avd.ra.24-00013

**Published:** 2024-03-15

**Authors:** Hiroko Morisaki

**Affiliations:** 1 Department of Medical Genetics, Sakakibara Heart Institute, Fuchu, Tokyo, Japan

**Keywords:** genetic testing, hereditary aortic aneurysm and dissection, Marfan syndrome, Loeys–Dietz syndrome, vascular Ehlers–Danlos syndrome

## Abstract

Hereditary aortic aneurysms and dissections, such as Marfan syndrome, differ in that they occur in younger patients without generally recognized risk factors, have a predilection for the thoracic rather than the abdominal aorta, and are at risk for dissection even at smaller aortic diameters. Early diagnosis, careful follow-up, and early intervention, such as medication to reduce aortic root growth and prophylactic aortic replacement to prevent fatal aortic dissection, are essential for a better prognosis. Molecular genetic testing is extremely useful for early diagnosis. However, in actual clinical practice, the question often arises as to when and to which patient genetic testing should be offered since the outcome of the tests can have important implications for the patient and the relatives. Pre- and post-test genetic counseling is essential for early intervention to be effective. (This article is a secondary translation of Jpn J Vasc Surg 2023; 32: 261–267.)

## Introduction

Aortic aneurysms and dissections commonly affect the abdominal aorta in elderly people and are attributed to underlying illnesses such as arteriosclerosis, hypertension, smoking, stress, hyperlipidemia, diabetes, and sleep apnea syndrome. It is considered a lifestyle-related disease.^1^^,^^2)^ On the contrary, Marfan syndrome and other types of hereditary aortic aneurysm and dissection differ in that they can develop even in young individuals without these risk factors and commonly affect the thoracic aorta, with even the aorta smaller in diameter being at risk of dissection.^3^^,^^4)^ In some hereditary aortic diseases, early initiation of medication, such as beta blockers and/or Angiotensin II receptor blockers (ARB), can reduce aortic root growth, and surgical interventions such as prophylactic aortic replacement can prevent dissection events; therefore, early diagnosis and early therapeutic intervention are recommended. To this end, genetic diagnosis by genetic testing provides decisive evidence for diagnosis. However, in actual clinical practice, the question of which patients and when genetic testing should be recommended frequently poses a problem. This paper reviews the clinical characteristics of typical hereditary aortic diseases and summarizes important points when performing genetic testing.

## Clinical Features of Hereditary Aortic Diseases

**Figure 1:**
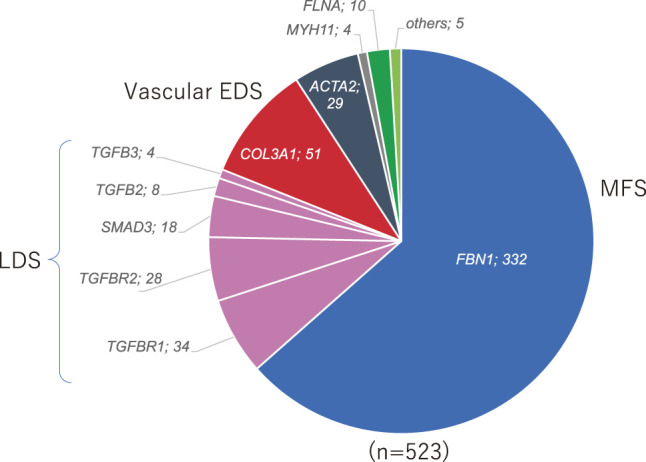
Fig. 1 Distribution of causative genes detected in patients with hereditary aortic aneurysm and dissection at our outpatient clinic of Medical Genetics (2005–2022). MFS: Marfan syndrome; LDS: Loeys–Dietz syndrome; EDS: Ehlers–Danlos syndrome

Most hereditary aortic aneurysms and dissections are associated with inborn defects caused by genetic abnormalities in some components that constitute the aortic wall, which is responsible for arterial wall weakness and the onset of aneurysms and dissections. They are broadly divided into syndromic hereditary aortic diseases with nonvascular lesions and nonsyndromic hereditary aortic diseases without nonvascular symptoms. In most cases of the former, systemic connective tissue damage occurs as a result of an abnormality in the extracellular matrix, which is the main constituent of connective tissue; therefore, characteristic symptoms tend to also be found in the bones, joints, skin, lungs, and eyes. A typical disease of this type is Marfan syndrome. Syndromic hereditary aortic diseases are often suspected based on characteristic clinical findings before the diagnosis is made. Conversely, in nonsyndromic hereditary aortic diseases, there are generally few physical symptoms other than vascular symptoms; therefore, suspicion arises from other kinds of information, such as onset age, histopathological features of the dissected vessel, and family history. Diagnosis is often confirmed when a mutation of the causative gene is found by genetic testing. As causative genes of syndromic aortic aneurysms and dissections in our cases, mutations of the *FBN1* gene responsible for Marfan syndrome, the transforming growth factor (TGF)-β-related genes responsible for Loeys–Dietz syndrome (LDS), and the *COL3A1* gene responsible for vascular Ehlers–Danlos syndrome (EDS) account for approximately 70%, 20%, and 10%, respectively. The *ACTA2* gene was the causative gene detected in approximately half of cases of nonsyndromic hereditary aortic aneurysms and dissections (**Fig. 1**). The following section provides a summary of the clinical characteristics of typical hereditary aortic diseases and introduces important points when performing genetic testing.

## Typical Hereditary Aortic Aneurysms and Dissections

### Marfan syndrome

Marfan syndrome is a systemic connective tissue disorder caused by a pathogenic mutation of the *FBN1* gene that encodes fibrillin-1, which is the main component of microfibrils constituting the extracellular matrix. It is a hereditary disease that is inherited in an autosomal dominant pattern. Fibrillin is not only the main constituent of elastic fibers but also involved in the regulation of TGF-β activity, which is considered important for maintaining the homeostasis of the extracellular matrix. Therefore, defects in fibrillin can cause aortic aneurysm and dissection, as well as various osteoarticular lesions.^5)^ Furthermore, microfibril is a primary component of Zinn’s zonule, which supports the lens of the eye; therefore, defective microfibril function can cause ectopia lentis. Diagnosis is determined using the revised Ghent diagnostic criteria (2010), which are international diagnostic criteria (**Tables 1** and **2**).^6)^

**Table:** Table 1 Revised Ghent nosology for Marfan syndrome (Loeys BL et al., J Med Genet 2010).^6)^

In the absence of FH
1. Aortic Root Dilatation(Z score ≥2)/Dissection, AND Ectopia Lentis=MFS*
2. Aortic Root Dilatation(Z score ≥2)/Dissection, AND FBN1 mutation=MFS
3. Aortic Root Dilatation(Z score ≥2)/Dissection, AND Systemic Score ≥7pts=MFS*
4. Ectopia lentis AND a FBN1 mutation associated with Aortic Root Dilatation=MFS
In the presence of FH
FH of MFS: a family member should be independently diagnosed using the above criteria (1–4)
5. FH of MFS, AND Ectopia lentis
6. FH of MFS, AND systemic score ≥7pts
7. FH of MFS, AND Aortic Root Dilatation(Z score ≥2 above 20 years old, ≥3 below 20 years old)

*Caveat: features suggestive of Shprintzen Goldberg syndrome, Loeys–Dietz syndrome, or vascular Ehlers–Danlos syndrome must be excluded and appropriate alternative genetic testing should be performed. MFS: Marfan syndrome; FH: family history

**Table:** Table 2 Scoring of systemic features for revised Ghent nosology (Loeys BL et al., J Med Genet 2010).^6)^

Wrist AND Thumb Sign	3
(wrist OR thumb sign)	(1)
Pectus carinatum deformity	2
(pectus excavatum deformity OR chest asymmetry)	(1)
Hindfoot deformity	2
(plain pes planus)	(1)
Pneumothorax	2
Dural ectasia	2
Protrusio acetabuli	2
Reduced US/LS AND increased arm/height AND no severe scoliosis	1
Scoliosis OR thoracolumbar kyphosis	1
Reduced elbow extension	1
Facial features (≥3 of 5 of the following 5 features)	1
(dolichocephaly, enophthalmos, downslanting palpebral fissures, malar hypoplasia, retrognathia)	
Skin striae	1
Severe myopia (>3 diopters)	1
Mitral valve prolapse (all types)	1

A score of ≥7 is considered a positive systemic score. US/LS: upper segment/lower segment ratio

In Marfan syndrome, the most important findings are aortic root lesions. Specifically, dissection of the ascending aorta caused by dilatation of the sinus of Valsalva or dilatation of the aortic root is most important. Dilatation of the sinus of Valsalva is defined as “an increase of 2 or more in the Z-score compared to the standard diameter calculated from age and body surface area.” However, in reality, calculating the “standard diameter from age and body surface area” is often difficult to determine because the definition of the calculation method is ambiguous. Commonly used calculation methods have been published on several websites, but there is no standardized method (e.g., https://marfan.org/dx/zscore-children/, https://marfan.org/dx/z-score-adults/, http://parameterz.blogspot.com/2008/09/aorticroot-z-scores.html). Furthermore, there are various measurement methods, including whether to use the systolic or diastolic phase, the inner edge or leading edge, and ultrasound or computed tomography (CT). Moreover, in childhood, even a slight measurement error has a major impact on the Z-score because the standard deviation is small. Thus, in actual clinical practice, diagnosis is determined comprehensively based on various factors, including the shape of the aortic root (pear-shaped dilatation), the balance of the aortic annulus diameter and the sino-tubular junction diameter, and dilatation speed. Furthermore, in echocardiography, characteristics of the cardiac valves, particularly those of the mitral valve, can be used as a reference, and Marfan syndrome is likely when findings such as mitral valve prolapse are observed.

For differential diagnosis, ectopia lentis (subluxation and luxation) has particularly high specificity, and Marfan syndrome is diagnosed based only on the two criteria of aortic lesions and ectopia lentis. In mild cases of ectopia lentis, only reduced visual acuity due to myopia or astigmatism is observed, which can be corrected with normal eyeglasses or contact lenses and thus be overlooked in general ophthalmologic examinations. Therefore, if suspected, slit lamp examination with mydriatic eye drops, such as a, is requested.

Most patients have a tall stature; in particular, patients characteristically have long limbs compared to the trunk, and they frequently voice an opinion that “I have trouble finding a long-sleeve shirt (that fits me).” Furthermore, while the height usually increases rapidly with a spurt during the growing years, many patients with Marfan syndrome have a tall stature since their infancy, and children with Marfan syndrome are clearly taller than other children of the same age.

Although not included in the diagnostic criteria, intraoral findings are also informative. In patients with Marfan syndrome, malalignment of the teeth and a high-arched palate are often seen because their jaw sizes or widths are small for their tooth sizes. The correction of malalignment of teeth caused by Marfan syndrome is covered by health insurance. Moreover, malalignment of the teeth often causes periodontal disease, and it is recommended to complete the treatment of periodontal disease before aortic surgery. Therefore, patients should be informed about the importance of dental hygiene when they are diagnosed with Marfan syndrome.

Skin striae (striae atrophicae) represent a finding associated with weakness of the subcutaneous tissue and are the same as stretch marks seen on the abdomen and thigh area in, for example, pregnant women and obese people; however, in Marfan syndrome, stretch marks are often found even without such conditions. Furthermore, stretch marks are characteristically seen at unusual sites such as the shoulders, upper arms, and back.

### LDS

LDS is the most important disease to differentiate from Marfan syndrome. The causative genes are TGF-β signaling-related genes, including *TGFBR1* and *TGFBR2*, and as in Marfan syndrome, it is a systemic connective tissue disorder inherited in an autosomal dominant pattern.^7^^,^^8)^ Some symptoms are similar to those in Marfan syndrome, including dilatation of the aortic root and skeletal lesions such as arachnodactyly. Other findings are characteristic of LDS, including ocular hypertelorism, bifid (cleft) uvula, and arterial tortuosity; however, cases without these findings are not uncommon. At present, a definite diagnosis requires a genetic diagnosis. Some emphasize that in LDS, “vascular lesions progress more rapidly and dissection occurs even in the aorta with a smaller diameter compared to Marfan syndrome”; however, there are significant differences among patients in both distribution and severity. Generally, when facial characteristics and osteoarticular symptoms are severe, cardiovascular symptoms also tend to progress rapidly, and when these nonvascular symptoms are mild, the course tends to be similar to that of typical nonsyndromic aortic aneurysm and dissection; as such, there may be some level of correlation between craniofacial severity and vascular severity. Furthermore, in addition to the abovementioned two genes, *SMAD3*, *TGFB2*, *TGFB3*, and *SMAD2* have been identified as causative genes to date; however, the clinical course somewhat differs depending on the causative gene, and in particular, the risk of dissection is not so high when the causative gene is any of the latter three genes (**Table 3**).^9^^–^^13)^ On the contrary, due care should be paid to the fact that descending aortic dissection can occur even if dilatation at the root is relatively mild, and careful monitoring is needed for lesions in the branching arteries as well as in the aorta.

**Table:** Table 3 Clinical features of Loeys–Dietz syndrome by associated gene (adapted from GeneReviews (ncbi.nlm.nih.gr/bookd/NBK1133))^20)^

Clinical feature	*TGFBR1*/*TGFBR2*	*SMAD3*	*TGFB2*	*TGFB3*	*SMAD2*
Developmental delay	−	−	−	−	−
Ectopia lentis	−	−	−	−	−
Cleft palate/bifid uvula	++	+	+	+	+
Widely spaced eyes	++	+	+	+	+
Craniosynostosis	++	+	−	−	−
Tall stature	+	+	++	+	+
Arachnodactyly	++	+	+	+	+
Pectus deformity	++	++	++	+	+
Clubfoot	++	+	++	+	−
Osteoarthritis	+	+++	+	+	+
Aortic root aneurysm	++	++	++	+	+
Arterial aneurysm	++	+	+	+	+
Arterial tortuosity	++	++	+	+	+
Early dissection	+++	++	+	+	+
Bicuspid aortic valve	++	+	+	+	+
Mitral valve insufficiency	+	+	++	+	+
Striae	+	+	+	+	+
Dural ectasia	+	+	+	−	−

−, absent; +, observed; ++, common; +++, more common

### Vascular EDS

Vascular EDS is an autosomal dominant hereditary disease presenting reduced type III collagen due to the *COL3A1* mutation, with weakness of organs, including the gastrointestinal tract, uterus, lungs, and muscles, as well as blood vessels.^14)^

Type III collagen is a homotrimer formed by three a1 chains; therefore, when one of the three chains becomes an abnormal peptide produced due to a missense mutation, normal trimers can no longer be formed. More specifically, in patients with a heterozygous missense mutation, the probability that trimers will be formed from three chains of normal peptides is (1/2)^3^ theoretically; thus, the normal collagen amount is 1/8. On the contrary, in null-type mutations, such as nonsense or frameshift mutations, the collagen amount decreases only by 50% because abnormal peptides are not produced. Therefore, patients with missense mutations are considered to be severe, and patients with null mutations are considered to be relatively mild. However, in reality, there are very large individual differences in symptoms, and the severity cannot be predicted by genotypes alone. Furthermore, although the syndrome is called “vascular,” weakness is observed in not just blood vessels but all tissues. In particular, initial complications often include nonvascular lesions such as gastrointestinal perforation, pneumothorax, and tendon rupture. Furthermore, many patients are misdiagnosed with coagulopathy because of the increased bleeding tendency caused by vascular fragility. At any rate, arterial dissection and aneurysm rupture are life-threatening lesions. Therefore, when the symptoms listed above are observed, vascular EDS should be suspected, and the diagnosis should be confirmed by genetic testing before fatal complications occur. If complications occur, appropriate treatment should be provided immediately. Furthermore, conventional catheter angiography not for therapeutic purposes should be avoided unless absolutely necessary. While extremely rare, similar symptoms are known to be caused by particular mutations in *COL1A1*, which encodes type 1 collagen.

### Nonsyndromic aortic aneurysm and dissection caused by the *ACTA2* gene

*ACTA2* encodes vascular smooth muscle actin, abnormalities of which cause dysfunction of smooth muscle cells in the aortic tunica media and, in turn, lead to aortic aneurysms and dissections. Except for some particular mutations,^15^^,^^16)^ *ACTA2* alterations manifest as nonsyndromic aortic aneurysms and dissections as lesions are limited to the vascular system.^17)^ Unlike Marfan syndrome and LDS, aortic dilatations caused by *ACTA2* gene mutations tend to occur in the ascending aorta and beyond rather than in the aortic root. Furthermore, type B dissections often develop while root dilatation is not observed. Therefore, management by routine follow-up with echocardiography, mainly of the ascending aorta, is insufficient, and other modalities, such as CT or magnetic resonance angiography, are performed additionally as needed.

## Genetic Testing

In recent years, remarkable progress has been made in genetic testing technology. While the cost directly involved in molecular analyses continues to decline, the accuracy of analyses has greatly improved, and genetic testing is used more often in the clinical diagnostic process. It is the advent of the so-called “genomic medicine” era. In particular, genetic tests for hereditary aortic diseases are often performed for early diagnostic purposes because, in most hereditary aortic diseases, the prognosis can be expected to improve with early diagnosis followed by early therapeutic intervention. In particular, in some diseases, the detection of pathogenic mutations (variants) by genetic testing is mandatory or almost mandatory for confirming the diagnosis (**Table 4**). However, several points require due care in terms of its actual usage. One point is how to think about the “pathogenicity” of the detected gene variant, and another is the question of when and in whom genetic testing should be provided.

**Table:** Table 4 Importance of genetic testing in diagnoses and management of patients with hereditary aortic aneurysm and dissection

Disease	Gene	Diagnostic significance	Significance for patient management
Marfan syndrome	*FBN1*	One of the diagnostic criteria	Early therapeutic intervention, determination of surgery timing
Loeys–Dietz syndrome	*TGFBR1, TGBFR2, SMAD3, TGFB2, TGFB3*, and *SMAD2*	Required to confirm diagnosis, subtype classification	Early therapeutic intervention, determination of surgery timing
Vascular Ehlers–Danlos syndrome	*COL3A1, (COL1A1)*	Required to confirm diagnosis	Affects patient management policy, useful for the identification of at-risk relatives
Familial thoracic aortic aneurysm and dissection	*ACTA2, MYH11, MYLK, PRKG1*, and *(LOX)*	Required to confirm diagnosis	Affects patient management policy, useful for the identification of at-risk relatives

### How to think about the “pathogenicity” of the gene variants

At present, the pathogenicity interpretation of the variants detected by genetic testing is conducted in accordance with the criteria of the American College of Medical Genetics and Genomics (ACMG).^18)^ In the ACMG/AMP guideline, the pathogenicity of the variant is classified into five categories according to an original scoring method based on evidence levels: “pathogenic,” “likely pathogenic,” variant of uncertain significance (VUS),” “likely benign,” and “benign.” These scoring criteria are strictly defined, and when a variant does not fully satisfy the criteria for pathogenic or benign, then the variant is considered “VUS.”

In other words, VUS implies that the variant only has insufficient information to determine its pathogenicity, and at this point in time, it cannot be deemed pathogenic or benign. It also means that in the future, the evaluation can change to “pathogenic” or “benign” if sufficient grounds for such judgment become available. This policy follows the basic idea that “diseases” should not be over diagnosed based solely on the results of genetic testing, and a variant is determined “pathogenic” very carefully. In fact, many gene variants reported as causative mutations have previously been downgraded to “VUS” upon re-evaluation. Furthermore, the ACMG/AMP guideline de-emphasized *in silico* pathogenicity prediction, which has been used as a basis for pathogenicity in many articles to date, and treated it as supporting evidence. Therefore, readers should know that new missense variants (the amino acid-related changes to a different amino acid) that have not been reported are basically considered to be “VUS” except for some. Furthermore, the ACMG/AMP guideline published in 2015 only presents a rough framework, and gene-specific ACMG/AMP classification criteria for individual genes are currently being reviewed by expert panels. At this point in time, many of them are being used as provisional criteria, and therefore, the judgment results for the same gene variants can differ depending on when the tests are done.

Moreover, in current genetic testing, it is standard practice to focus the analyses on the exons, the protein-coding regions of the genome; however, it has been known that pathogenic variants in the non-exonic regions called the introns can be responsible for splicing abnormalities. To detect such mutations, analysis using tissue-derived mRNA is required, and it is likely that such mutations will be overlooked in routine analysis of genomic DNA alone. In other words, in general, genetic testing result reports indicating “no pathogenic variant” will be returned. Previously reported pathogenic intronic variants have been increasingly reflected in the updated probe design for genome analysis; however, it is almost impossible to detect new variants. Thus, readers should know that “some causative mutations cannot be detected for technical reasons, even if they exist.”

Thus, when genetic testing results are disclosed, the results should be explained after these circumstances are understood. In particular, when VUS results come back, a higher priority should be given to clinical diagnosis, and genetic information should be treated as reference information; efforts should also be made to always obtain the latest information from public databases, such as ClinVar (https://www.ncbi.nlm.nih.gov/clinvar/) and other resources. Ultimately, judgment should be determined comprehensively based on the clinical information of each patient and the information of their family in principle.

### When and to whom should genetic testing be provided

The prognosis of hereditary aortic diseases can be expected to improve with early diagnosis and early therapeutic intervention, and therefore, genetic testing for early diagnosis is proactively used in clinical settings. In Japan, genetic analysis for major hereditary aortic diseases has been covered by health insurance since April 2016 and is now available as a routine outsourced test. Nonetheless, genetic testing should be performed only when sufficient benefit can be expected after careful thinking with the first priority placed on benefit for the patients and their family members, keeping in mind the following basic points: “genetic information basically does not change throughout one’s life” and “genetic information is shared among blood relatives.” Furthermore, the financial aspects may need to be considered in some cases. According to the National Health Insurance system, costs for genetic testing of hereditary aortic diseases are 24,000 Japanese yen, with a 30% copayment. However, out-of-pocket expenses can be reduced through the effective use of various financial support systems for medical care, such as the welfare system for the physically disabled after cardiac valve replacement and the pediatric medical expense subsidization scheme of each local government.

When implementing genetic testing, the “Guidelines for Genetic Tests and Diagnosis in Medical Practice” (revised March 2022)^19)^ should be observed; it is important to note that “it is recommended to obtain written consent after providing a thorough explanation and support. As a rule, the attending physician will verify the explanation before these genetic tests and consent/assent (informed consent for adults and informed assent for minors). Furthermore, arrangements should be made so that patients can receive genetic counseling by an expert and support for decision-making as needed.” Moreover, when performing genetic tests for minors, “assent should be obtained from a representative who can provide assent to testing on behalf of the minor concerned; in such instances, due care should be paid to the subject’s best interest. Furthermore, an explanation appropriate for the subject’s level of understanding should be provided, and it is preferable to obtain assent from the individual concerned (informed assent)”.

In addition, genetic testing of the proband (the person diagnosed first in a family line) has great significance in that it can lead to the early diagnosis of blood relatives. On the contrary, “when” and “to whom” to provide genetic testing should be carefully considered for blood relatives. In particular, for individuals remaining asymptomatic, due care should be paid to the fact that genetic testing of asymptomatic individuals is limited to cases in which preclinical diagnosis is expected to be very beneficial for health management. However, this assessment of being “beneficial” differs for each disease and each patient. In the case of Marfan syndrome, as an example, many patients with Marfan syndrome have skeletal symptoms from early childhood, but the progression of aortic lesions occurs in later childhood or even later; thus, in Japan, genetic testing is performed mainly when the patient has symptoms suggestive of the disease and a definitive diagnosis is needed for the management of or therapeutic intervention. However, in Europe and the United States of America, when a parent is known to have a genetic mutation, it is preferred to start the β-blocker treatment as early as possible if his/her child carries the mutation, and genetic testing is recommended even for asymptomatic children and infants to clarify their inherited risk. However, readers should know that presymptomatic diagnosis in children can occasionally lead to excessive interference and restrictions to the daily life of the affected child and changes in the relationships with surrounding individuals.

Accordingly, in actual practice, the advantages and disadvantages associated with testing should be thoroughly explained during genetic counseling before tests. Evidently, tests should be performed only when testing is considered to be sufficiently beneficial for the subject. After testing, continued follow-up is needed so that the subject and his/her family members can use the genetic test results effectively in the future.

## Additional Remarks

This paper was presented at the 35th educational seminar of the Annual Meeting of the Japanese Society for Vascular Surgery (October 29, 2022, Yokohama).

Genetic analyses have been approved by the ethical review board, and consent has been obtained from all patients for genetic analysis as research. National Cerebral and Cardiovascular Center (before March 2016): “Genetic analysis to elucidate the pathogenesis of familial arterial diseases such as Marfan syndrome” (M14-20), Sakakibara Heart Institute (after August 2016): “Genetic analysis to elucidate the pathogenesis of young-onset and familial arterial diseases such as Marfan syndrome” (16-035).

## Disclosure Statement

The author has no conflicts of interest to declare.
